# Optimized Machine Learning Pipeline for Lung Cancer Classification: Feature Reduction and Hyperparameter Tuning

**DOI:** 10.3390/diagnostics16081198

**Published:** 2026-04-17

**Authors:** Gufran Ahmad Ansari, Salliah Shafi, Lamees Alhazzaa

**Affiliations:** 1 College of Computer and Information Sciences, Imam Mohammad Ibn Saud Islamic University (IMSIU), Riyadh 13318, Saudi Arabia; gsansari@imamu.edu.sa (G.A.A.); lahazzaa@imamu.edu.sa (L.A.); 2 School of Computational Science, GNA University, Sri Hargobindgarh, Phagwara-Hoshiarpur Road, Phagwara 144401, India

**Keywords:** lung cancer classification, feature selection, hyperparameter tuning, metaheuristic optimization, machine learning

## Abstract

**Background:** Lung cancer remains one of the leading causes of cancer-related mortality worldwide, primarily due to late diagnosis. Although machine learning (ML) techniques have been widely applied for lung cancer classification, many studies lack a fully optimized end-to-end pipeline using routine clinical data. This study proposes an optimized ML framework that integrates demographic, lifestyle, and clinical features with systematic hyperparameter tuning to improve classification performance. **Methods:** A dataset of 309 patient records containing demographic, lifestyle, and clinical attributes was used. The data were preprocessed and split into training and testing sets in an 80:20 ratio. Feature selection was performed using metaheuristic algorithms, including Red Deer Optimization, Binary Grasshopper Optimization, Gray Wolf Optimization, and Bee Colony Optimization. Six ML classifiers—Logistic Regression, Support Vector Classifier, Gradient Boosting, Random Forest, K-Nearest Neighbors, and Gaussian Naive Bayes—were trained with optimized hyperparameters. Model performance was evaluated using accuracy, precision, recall, F1-score, and ROC–AUC. **Results:** The optimized pipeline significantly improved classification performance. Logistic Regression achieved the highest accuracy of 91.07% with an AUC of 0.91, outperforming more complex ensemble models. Gradient Boosting and Random Forest both achieved an accuracy of 87.5%, while other classifiers demonstrated moderate performance. **Conclusions:** The proposed optimized ML pipeline enhances lung cancer classification accuracy using routine clinical data. The results highlight that simpler, well-optimized models can outperform complex approaches on structured datasets. This framework shows strong potential for early lung cancer risk screening and clinical decision support, although further validation on larger datasets is recommended.

## 1. Introduction

Lung cancer (LC) remains one of most prevalent and deadly forms of cancer globally, accounting for a substantial proportion of annual cancer-related deaths. The challenge of accurately predicting and detecting lung cancer at an early stage has led to extensive research into the use of machine learning (ML) algorithms to enhance diagnostic capabilities and patient outcomes. The advancement of sophisticated computational techniques has opened new avenues for analyzing complex medical data, contributing significantly to improvements in cancer classification and prognosis [1]. The human body is made up of trillions of cells that normally grow and divide to form new cells. As time goes on, old or damaged cells die and new ones take their place. Sometimes, this natural process does not work properly. When that happens, damaged or abnormal cells may continue to grow without control. These cells can form tumors, which may be cancerous (malignant) or non-cancerous (benign) [2]. Cancerous cells can spread to nearby tissues and even to other parts of the body through a process called metastasis. Lung cancer is mainly caused by genetic changes in cells. However, external factors like smoking, second-hand smoke exposure to harmful gases and air pollution can also trigger these changes. These factors interfere with the normal cell cycle, causing damaged cells to grow rapidly instead of dying, which leads to the development of cancerous tumors [3]. LC is one of most common and deadliest types of cancer worldwide. As per the World Health Organization (WHO), out of 10 million cancer cases reported in 2020, about 2.21 million were lung cancer cases. Around 1.8 million people die from lung cancer every year, making it the leading cause of cancer-related deaths [4]. Lung cancer progresses through four stages. In the early stages (I and II), it is often treatable and potentially curable, but symptoms are typically absent. Clinical symptoms usually emerge in advanced stages (III and IV), where malignant tumors exhibit aggressive growth and may invade surrounding organs and tissues. In contrast, benign tumors grow slowly and can often be removed with surgery therapy or other medical treatments [5]. Machine learning (ML) has become an essential tool in the classification and diagnosis of LC, offering significant improvements in accuracy, speed and decision-support compared to traditional medical techniques. A robust ML pipeline for lung cancer classification typically includes data preprocessing, feature extraction, feature reduction, model training, hyperparameter tuning, and performance evaluation. Feature reduction methods like Principal Component Analysis (PCA) and Recursive Feature Elimination (RFE) help in minimizing noise and dimensionality by selecting only the most relevant features from clinical data, medical imaging, or genomic datasets. Once features are optimized, several MLAs can be applied to classify lung cancer cases, including Logistic Regression (LR), Support Vector Classifier (SVC), Random Forest Classifier (RFC), Gradient Boosting Classifier (GBC), Gaussian Naive Bayes (GNB), and K-Nearest Neighbors Classifier (KNC). Each algorithm offers unique advantages: LR is simple and interpretable for binary classification; SVC performs well in high-dimensional spaces; RFC and GBC, as ensemble models, provide robustness and high predictive accuracy; GNB is fast and suitable for probabilistic classification and KNC is easy to implement and effective for small-scale datasets [6,7]. These models are further refined through hyperparameter tuning techniques such as Grid Search or Random Search to ensure optimal performance. Model evaluation is conducted using metrics like accuracy, precision, recall, F1-score and ROC–AUC to assess their effectiveness in detecting and classifying lung cancer stages or tumor types. Studies have shown that ensemble models like RFC and GBC often outperform others due to their ability to handle complex and imbalanced data. When integrated with clinical records or radiological images, this machine learning pipeline can accurately distinguish between benign and malignant tumors, predict the stage of lung cancer and support personalized treatment strategies. Ultimately, ML-based systems contribute significantly to early diagnosis, reduce diagnostic errors and aid healthcare professionals in making timely and informed clinical decisions [8]. This study is centered on LC, a condition that has been extensively examined through the lens of ML in recent years. A wide range of research efforts have applied ML techniques to analyze, predict and classify lung cancer cases. In this work, a comprehensive methodology is proposed for building accurate ML classification models capable of predicting the likelihood of lung cancer occurrence. The models are trained using a set of input features derived from commonly observed risk factors, lifestyle habits (such as smoking), and early symptoms [9]. By leveraging these features, the proposed ML framework aims to assist in the early detection and diagnosis of lung cancer, thereby contributing to improved outcomes and timely medical intervention.

The proposed model is aimed at being a clinical decision-support instrument of early lung cancer risk screening and referral triage based on routine clinical and lifestyle data. It helps clinicians determine high-risk patients who may need an early referral to imaging or specialist examination as an addition and not a substitute to conventional diagnostic methods. The paper has been modified to explain this clinical application. The main objectives of this study are:

- To design an optimized machine learning pipeline for accurate lung cancer classification using clinical data.
- To apply effective data preprocessing techniques (such as handling missing values, normalization, and noise reduction) to improve data quality and model performance.
- To perform feature selection to identify the most relevant clinical attributes and enhance classification accuracy.
- To evaluate and compare multiple machine learning classifiers, including Logistic Regression (LR), Support Vector Classifier (SVC), Gradient Boosting Classifier (GBC), Random Forest Classifier (RFC), K-Nearest Neighbors (KNNs), and Gaussian Naive Bayes (GNB).
- To assess the performance of these models using standard evaluation metrics such as accuracy, precision, recall, F1-score, and ROC–AUC.
- To identify the most reliable and robust classifier for supporting the early and accurate diagnosis of lung cancer.
- To establish a scalable and extensible framework that can be enhanced in future research through the integration of advanced deep learning techniques.

Unlike prior studies that focus on isolated optimization strategies or imaging-based deep learning models, this work proposes a unified and clinically interpretable machine learning pipeline that jointly integrates metaheuristic feature selection and hyperparameter optimization using routine clinical data for early lung cancer risk screening.

### Research Gap

Despite extensive research on lung cancer prediction using machine learning, several gaps still remain. Most existing studies apply individual or ensemble classifiers without a fully optimized end-to-end pipeline, often relying on manual or limited feature selection and minimal hyperparameter tuning, which can lead to suboptimal performance. Many works emphasize imaging-based deep learning models, while comparatively less attention is given to optimized machine learning frameworks using easily accessible clinical and lifestyle data for early screening. In addition, the fair comparison of multiple classifiers under a unified, feature-optimized framework and comprehensive evaluation using robust performance metrics are still lacking. This study addresses these gaps by proposing an optimized machine learning pipeline that integrates metaheuristic-based feature selection and automated hyperparameter tuning to improve reliability and diagnostic accuracy.

The rest of this paper is organized as follows. Section 1 provides an introduction to lung cancer, the motivation for using machine learning techniques in early diagnosis, the objectives of the study, and the identified research gap. Section 2 reviews related work and summarizes existing machine learning, ensemble, and optimization-based approaches to lung cancer prediction and classification. Section 3 describes the proposed methodology, including data collection, exploratory data analysis, preprocessing, applied machine learning models, metaheuristic-based feature selection techniques, hyperparameter tuning and evaluation metrics. Section 4 presents the experimental results and discusses the comparative performance of the proposed models using standard performance measures. Finally, Section 5 concludes the paper by highlighting the main findings and contributions, and outlines future research directions, including the integration of deep learning and explainable AI techniques.

## 2. Literature Review

LC is a condition characterized by the uncontrolled growth of cells in lung tissue and is leading cause of death among both men and women [10]. The use of ML and ensemble learning techniques for cancer prediction has been a major focus of research, with numerous methods applied across different types of cancer. Conventional MLAs have been extensively utilized in predicting various cancers, including lung cancer, breast cancer and cervical cancer. Numerous researchers have explored ensemble learning techniques to enhance the prediction accuracy of cancers such as lung, breast and cervical cancer by applying these methods to both medical imaging and clinical data. In the context of lung cancer prediction, ref. [11] implemented bagging and randomized node optimization, taking into account key risk factors such as genetic predisposition, air pollution, smoking and various clinical symptoms. Ref. [12] utilized five ensemble techniques, including bagging and AdaBoost (ADB) on the dataset to predict lung cancer cases. Ref. [13] introduced an ensemble learning model combining Random Forest (RF) with a self-paced learning bootstrap which effectively integrated both high- and low-quality data samples to improve prognosis prediction. Ref. [14] applied bagging-based ensemble models using Random Forest to forecast patient survival, proposing an ML pipeline tailored to imbalanced datasets. In a comparative study, ref. [15] evaluated several ensemble approaches such as XGBoost (XGB), LightGbm (LGBM), bagging, and AdaBoost with XGB, achieving the highest prediction accuracy of 74.42% for lung cancer detection [15]. Similarly, in ref. [16], researchers employed various classification algorithms, including Neural Networks, Radial Basis Function Networks (RBFNs), Support Vector Machines (SVMs), Logistic Regression, Random Forest, J48, Naive Bayes and K-Nearest Neighbors to predict LC. Among these, the RBFN model delivered the highest accuracy, achieving 80.25% on the lung cancer dataset. Furthermore, the primary aim of the study in [17] was the early detection of LC by evaluating the effectiveness of multiple classification techniques. The authors tested models such as Naive Bayes, SVM, Decision Trees and Logistic Regression. When applied to the UCI lung cancer dataset, Logistic Regression produced the best performance, with an accuracy of 86.9%. In contrast, on the lung cancer dataset, SVM achieved a slightly higher accuracy of 87.2% from the data [18]. In the area of breast cancer research, ref. [19] proposed an improved ensemble learning method called Improved XGBoost (I-XGBoost), which integrates big data analytics to enhance breast cancer prediction and diagnosis. Their study emphasizes the selection of the most relevant features for accurately classifying tumors as either malignant or benign. The findings show that the XGB model achieved an impressive accuracy of 99.84% when implemented using Spark’s. In a separate study, ref. [20] developed a predictive model for breast cancer by incorporating multifactorial data along with optimization techniques. Their approach utilized a combination of demographic, laboratory and mammographic data consisting of 5178 independent records and 24 distinct features, thereby considering a broad range of factors that could impact the accuracy of breast cancer diagnosis [21]. Among the various models tested, the Random Forest (RF) algorithm achieved the highest accuracy of 80%. Despite its high sensitivity of 85%, the Area under Curve (AUC) model’s score was only 56%, suggesting limited generalization capability on unseen data. In a related study, ref. [22] assessed several MLAs for breast cancer prediction using the WDBC dataset. Their analysis included both traditional and ensemble models such as Naive Bayes (NB), Logistic Regression (LR), Support Vector Machine (SVM), K-Nearest Neighbors (KNNs), Decision Tree (DT), Random Forest (RF), AdaBoost (ADB) and XGBoost (XGB). Among these, XGBoost emerged as the top-performing model, achieving an impressive accuracy of 67% and an AUC score of 59.9%. However, the study did not provide an in-depth discussion on hyperparameter tuning, nor did it explore the role of feature engineering, which could have further enhanced the model’s performance [23]. A comparative study evaluated the MLM Support Vector Machine (SVM), Random Forest (RF), Logistic Regression (LR), Decision Tree (DT), and K-Nearest Neighbors (KNNs) for breast cancer prediction. Among these, SVM demonstrated the best performance, achieving an accuracy of 77.2%, a precision of 78%, a recall of 84% and an AUC of 96.6%. The study highlights the potential value of incorporating explainable AI (XAI) techniques to enhance the interpretability of results, which is essential for adoption. In a separate study, ref. [24] introduced an enhanced version of the XGBoost algorithm, named EXSA, tailored for survival analysis. This model is specifically designed to manage tied events and predict disease progression in breast cancer patients using clinical data from the CRCB at West China Hospital, Sichuan University [25]. The performance of EXSA was compared with other models, including the Cox Proportional Hazards model, Random Survival Forest (RSF), and Gradient Boosting (GB), using metrics such as the concordance index (C-index) and time-dependent AUC. While EXSA showed promising results, its complexity and limited focus on model interpretability could hinder its practical use in real-world clinical settings [26]. In the domain of cervical cancer prediction, ref. [27] proposed a comprehensive approach, leveraging various MLAs, including SVM, RF, KNN, DT, NB, LR, ADB, GB, MLP, Nearest Centroid Classifier (NCC) and an ensemble voting method. To improve predictive performance, the study adopted a hybrid feature selection framework that combines Principal Component Analysis (PCA), Select Best and XGBoost-based feature ranking. This hybrid strategy was used to extract the most informative and discriminative features from clinical and behavioral data associated with cervical cancer risk. Among the models evaluated, the ensemble voting classifier, which combined RF and MLP, delivered the highest performance, with an accuracy of 79% in RF and a precision, recall and F1-score of 76% each [28]. This result highlights the advantage of ensemble approaches in handling the complexity and variability of medical datasets. Moreover, the study underscores the importance of robust feature selection, particularly in healthcare domains, where noisy or irrelevant features can significantly impact the accuracy and reliability of predictions. While the results are promising, research also points to potential improvements. One key direction for future work involves the integration of explainable AI (XAI) techniques. By adding interpretability to model outputs, XAI can help clinicians and healthcare professionals understand the reasoning behind predictions, identify critical risk factors and tailor prevention or intervention strategies more effectively [29]. In particular, incorporating XAI could provide valuable insights into how specific behavioral, demographic or physiological features such as those linked to obesity, lifestyle habits, or reproductive history contribute to cervical cancer risk. Such transparency would be essential for building trust in AI-driven diagnostic tools and ensuring their responsible and effective use in clinical practice [30]. Recent advancements in research have furthered the development of predictive models for LC management. One such approach involved handling imbalanced datasets by employing MLAs like XGBoost, Logistic Regression and Random Forest, where the RF model achieved a commendable accuracy of 68% [31]. In related studies, the integration of AdaBoost with Convolutional Neural Networks (CNNs) for analyzing CT scan images resulted in significant improvements in predictive accuracy. Additionally, use of Chi-square-based feature selection techniques, combined with various machine learning models, led to better classification performance [32]. Among these, AdaBoost has consistently demonstrated superior results in terms of both accuracy and AUC, particularly when applied to large-scale datasets. To enhance LC prognosis, researchers have utilized blood test data in combination with ML models such as RF, achieving high levels of accuracy, recall, and AUC [33]. Multiple datasets, including those from Kaggle and Lanzhou University, were used in a study to strengthen the prediction and classification of LC [34]. The authors propose a hybrid genetic–fuzzy algorithm for LC detection. However, a major limitation of this approach is the small size of the dataset, which may result in inaccurate feature selection. Similarly, ref. [35] introduced a computer-aided diagnosis (CAD) method for identifying malignant cancer types, but its effectiveness may be limited when applied to new data due to the restricted training dataset. Table 1 provides a detailed summary of these methodologies in the form of a comprehensive literature review. Research on lung cancer detection has utilized various computer-aided diagnosis (CAD) techniques. However, limited work has been done on optimizing the Random Forest (RF) hyperparameter, primarily because the process is time-consuming. The manual tuning of the hyperparameter requires considerable effort and time. To address these limitations, we proposed an automated hyperparameter optimization model. The effectiveness of both the proposed and existing optimization methods was evaluated using performance metrics such as accuracy, recall, weighted average and F1-score. Table 1 shows the comparison below.

## 3. Methodology

**Figure 1:**
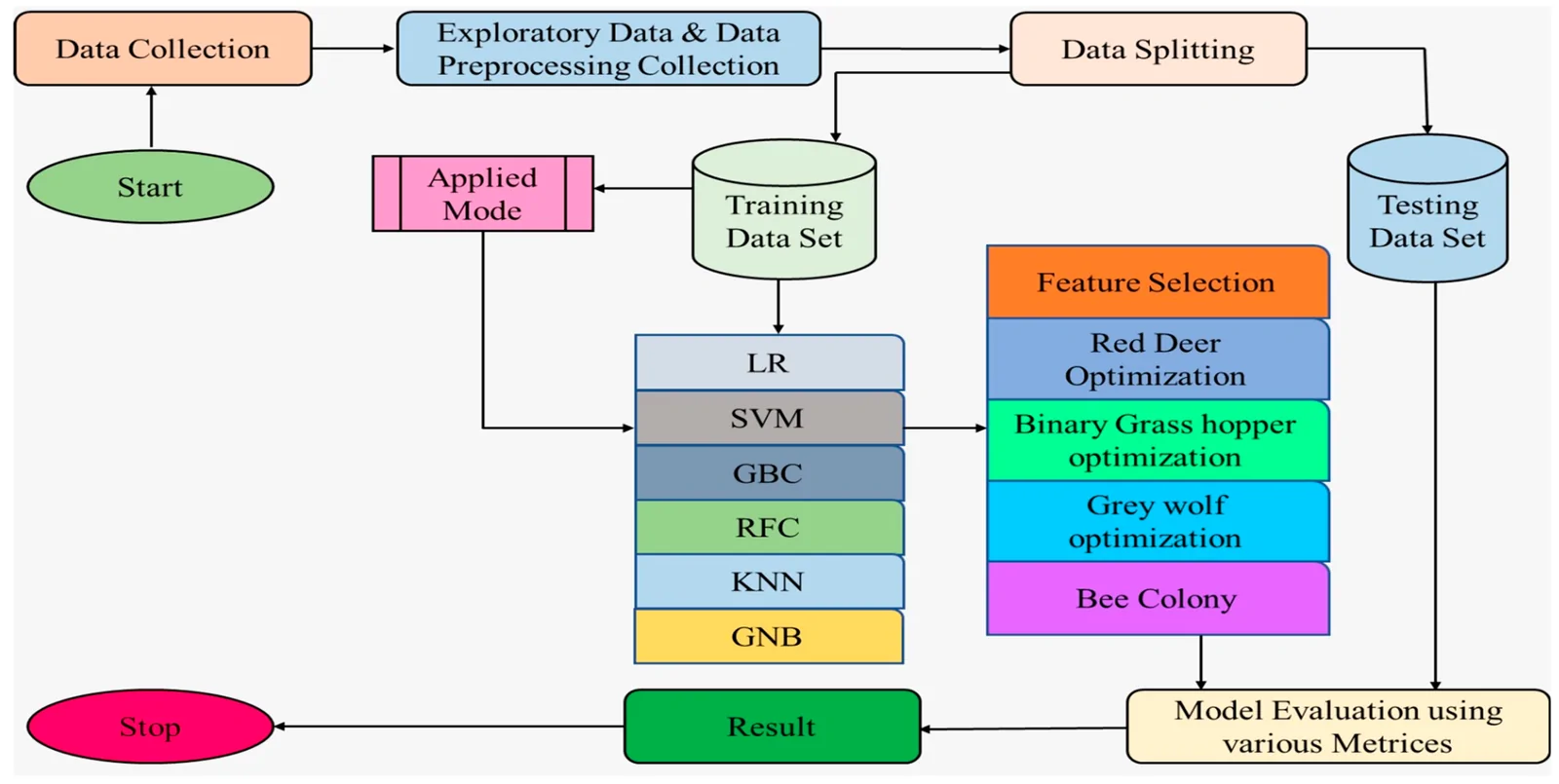
Comprehensive workflow.

The proposed pipeline represents a sequential and modular architecture designed to ensure systematic model development, reproducibility and minimized data leakage. Figure 1 presents a comprehensive workflow for lung cancer prediction using machine learning (ML) models enhanced with nature-inspired optimization algorithms. The process initiates with data collection, which is then subjected to exploratory data analysis and preprocessing to clean and transform the data for improved model training. After preprocessing, the dataset is split into training and testing sets. The training dataset is used to train several ML classifiers, including Logistic Regression (LR), Support Vector Machine (SVM), Gradient Boosting Classifier (GBC), Random Forest Classifier (RFC), K-Nearest Neighbors (KNNs), and Gaussian Naive Bayes (GNB). These models are applied in the Applied Mode phase to evaluate their baseline performance. To enhance model accuracy and reduce dimensionality, feature selection techniques are incorporated using metaheuristic optimization algorithms, such as: Red Deer Optimization, Binary Grasshopper Optimization, Gray Wolf Optimization and Bee Colony Algorithm. These optimization algorithms help in selecting the most relevant features from the dataset, which improves the predictive capability and reduces computational overhead. Optimized models are then tested on the testing dataset, and model evaluation is carried out using various performance metrics such as accuracy, precision, recall, and F1-score. The final results from the best-performing models are collected and the process concludes with the stop phase. This framework ensures a structured and effective pipeline for improving lung cancer detection using hybrid ML and bio-inspired optimization techniques. In this study, the term pipeline refers to a structured end-to-end machine learning workflow integrating preprocessing, metaheuristic-based feature selection, hyperparameter tuning, model training, and evaluation within a unified framework.

### 3.1. Data Collection

The dataset used in this study was compiled from 309 patient records containing both demographic and clinical attributes relevant to lung cancer risk. As shown in Table 2, the dataset includes 11 input features: gender, age, smoking habits, yellow fingers, anxiety, peer pressure, chronic disease, fatigue, allergy, wheezing, and alcohol consumption. These attributes were selected because they capture a combination of lifestyle factors (such as smoking, alcohol use, and peer pressure), clinical indicators (wheezing, chronic disease, fatigue) and personal history (age, gender, allergies). Data values were recorded in binary format (0 = No, 1 = Yes) for categorical features, while age was retained as a numeric variable. The target attribute indicates whether a patient was diagnosed as benign (0) or malignant (1), making the dataset suitable for a binary classification task. Prior to analysis, missing values were addressed, variables were encoded, and continuous features were normalized to ensure consistency across the dataset. This structured collection of attributes ensures a comprehensive view of lung cancer risk factors and supports the development of robust machine learning models for early and accurate classification [1].

The dataset was preprocessed to handle missing values, encode categorical attributes, and normalize continuous variables. The target variable distinguishes between benign and malignant cases, making the dataset well-suited for a binary classification problem. Its structured format ensures a comprehensive representation of risk factors and enables the development of accurate machine learning models for lung cancer detection.

### 3.2. Exploratory Data Collection and Preprocessing

**Figure 2:**
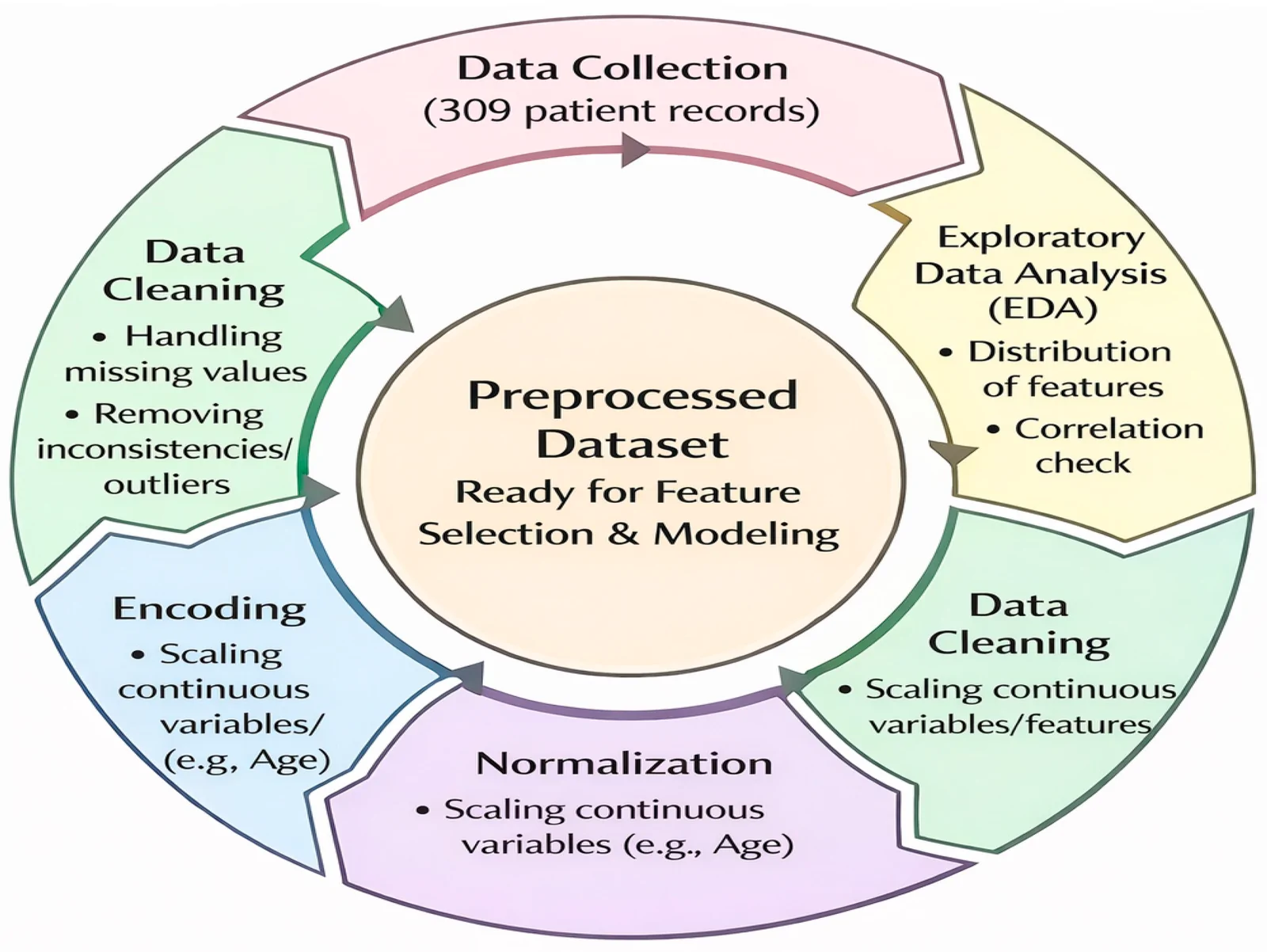
Data preprocessing workflow.

The workflow outlines the sequential steps undertaken to prepare the lung cancer dataset for ML. Data were obtained from 309 patient records encompassing demographic, lifestyle, and clinical risk factors. During the exploratory data analysis (EDA) stage, feature distributions, correlations, and potential class imbalances were examined. Data cleaning was then performed to address missing values, inconsistencies, and outliers. Categorical attributes such as smoking, wheezing, and alcohol consumption were encoded into binary format, while continuous variables, including age, were normalized to ensure consistency. The final outcome was a well-structured and reprocessed dataset optimized for feature selection and classification modeling, as depicted in Figure 2. The dataset was divided into two subsets using an 80:20 split ratio. Eighty percent of the data was allocated to the training set, which was used for test and optimizing the model, while the remaining twenty percent was kept aside as a testing set to evaluate model performance. This approach ensured that the model was assessed on unseen data, helping to provide a reliable estimate of its generalization ability and reducing the likelihood of overfitting.

In the training set, usually 70–80% of the data is used to model patterns, relationships and rules within the data. During this phase, the model adjusts its parameters to minimize prediction errors. In the testing set, typically 20–30% of the data is kept separate and is used only after training to evaluate model performance on unseen data. This helps measure the model’s ability to generalize, ensuring it performs well, not just on the training data, but also on new, real-world data.

#### 3.2.1. Statistical Validation

Statistical validation has been incorporated by reporting ROC–AUC analysis and confusion matrix-derived measures (precision, recall, F1-score). In addition, k-fold cross-validation has been applied to reduce sampling bias, and mean ± standard deviation of performance metrics is now reported to assess result stability. These additions provide a statistically more reliable evaluation of the proposed model. Additionally, ROC–AUC analysis and confusion matrix-based metrics (precision, recall, and F1-score) were included to ensure a statistically reliable evaluation of the proposed model [1].

#### 3.2.2. Hyperparameter Tuning

Hyperparameter tuning was performed to optimize the performance of all machine learning classifiers used in this study. Instead of relying on default parameter settings, a systematic search strategy was applied to identify the most suitable hyperparameter combinations for each model. Grid Search with k-fold cross-validation was employed on the training dataset to ensure fair comparison and reduce overfitting. For Logistic Regression, parameters such as the regularization strength and solver type were optimized. For Support Vector Classifier, the penalty parameter and kernel settings were tuned. Ensemble models, including Random Forest and Gradient Boosting, were optimized by adjusting the number of estimators, tree depth, and learning rate. For K-Nearest Neighbors, the number of neighbors and distance metric were tuned, while Gaussian Naïve Bayes was evaluated with optimized variance smoothing. The final model configurations were selected based on cross-validated performance and subsequently evaluated on the independent test set [42]. To ensure that the proposed pipeline does not rely on default model configurations, systematic hyperparameter optimization was performed for all classifiers using Grid Search combined with k-fold cross-validation (k = 5). The optimal hyperparameters were selected based on the mean cross-validated ROC–AUC score. This process reduces overfitting, improves generalization capability, and ensures fair comparison among classifiers under an optimized setting.

### 3.3. Applied Models and Evaluation Protocol

This paper has used six machine learning classifiers, namely, Logistic Regression (LR), Support Vector Classifier (SVC), Gradient Boosting Classifier (GBC), Random Forest Classifier (RFC), K-Nearest Neighbors (KNNs) and Gaussian Naïve Bayes (GNB), to classify lung cancer. Considering that the dataset size (309 samples) is relatively small, specific attention was paid to significant overfitting reduction and the strength of performance estimation. We implemented six popular and clinically interpretable machine learning models, which were used to categories the lung cancer risk based on commonly used routine demographic, lifestyle, and clinical characteristics. The chosen models are a compromise between predictive accuracy, interpretability and computational performance, which is critical in real-world clinical decision-support systems. The use of Logistic Regression (LR) was because it is easy to use and highly interpretable. It approximates the likelihood of lung cancer occurrence in order to be based on a linear product of input risk variables, which is especially suitable in the clinical environment, in which it is important to learn the contribution of each variable. Clinicians are able to directly interpret model coefficients in order to determine the effect of factors on the risk of cancer, including smoking history, age, or respiratory symptoms. The Support Vector Classifier (SVC) was used to model possible non-linear associations among possible risk factors and outcomes of lung cancer. Compared to LR, SVC has the ability to divide high-risk and low-risk groups of patients in moderately complex clinical data. The ensemble learning tools included Gradient Boosting Classifier (GBC) and Random Forest Classifier (RFC), which is a combination of numerous Decision Trees to enhance predictive accuracy and robustness. These are more appropriate models that can be used to model complex interactions between clinical variables, and are also model-less to noise. The importance of features obtained with these models can inform the proportionate contribution of risk factors. A simple, instance-based classifier, which assigns risk depending on similarity to the already-detected patient profiles, was used as the K-Nearest Neighbors (KNNs). Although simple to apply, it relies on the data distribution and sample, which is why its performance can be used on a limited scale only as a baseline comparison. The reason behind the inclusion of Gaussian Naïve Bayes (GNB) is its computational efficiency and appropriateness with small clinical data. In spite of its simplifying assumptions, GNB can be effective in medical classification and can generate probabilistic results, which might be useful in risk stratification. On the whole, models were chosen to present the varying degrees of complexity and interpretability. Their relative analysis in the context of a common and optimized pipeline enables them to evaluate trade-offs between accuracy and clinical usability, with a specific focus on models with the potential to provide the early risk screening of lung cancer with the help of simple clinical data. To be consistent with similar studies, the first evaluation of the model was performed with an 80:20 train–test split. Moreover, cross-validation at the k-fold was also used in the hyperparameter tuning to minimize bias due to only one random partition as well as enhance the consistency of the selected models. The mean and standard deviation of performances are presented as (mean) folds where necessary. Though several metaheuristic optimization algorithms were studied to carry out the feature selection, the performance difference obtained across the classifiers is small. These reported improvements, therefore, should be viewed as a trend, in our view, rather than statistically conclusive measures of being superior. No formal tests of statistical significance or confidence intervals were calculated and all results of observed differences in accuracy may be in part because of the sampling variability that is inherent in small datasets [43]. The selected classifiers represent a balance between interpretability (Logistic Regression), robustness (Random Forest, Gradient Boosting), and computational efficiency (KNN, Naïve Bayes). Metaheuristic-based feature selection was employed to identify clinically relevant risk factors, reduce redundancy, and improve diagnostic reliability, which is critical for early lung cancer screening using limited clinical data. Each model was selected by considering the dataset characteristics, including the relatively small sample size (309 records), predominantly binary feature distributions, low missingness after preprocessing, and binary outcome prevalence. Simpler and interpretable models such as Logistic Regression and Gaussian Naïve Bayes were favored for their robustness and reduced overfitting risk on small datasets, while ensemble methods (Random Forest, Gradient Boosting) were included to capture potential non-linear feature interactions. This alignment between model choice and data properties clarifies the methodological rationale and supports reliable performance evaluation.

### 3.4. Feature Selection (FS)

In this study, feature selection was performed using metaheuristic optimization techniques to identify the most clinically relevant risk factors contributing to lung cancer classification. Rather than focusing on mathematical formulations, these algorithms function as intelligent search strategies that evaluate different combinations of features and retain those that maximize predictive performance (accuracy and ROC–AUC). As shown in Table 3, the selected features primarily included smoking history, wheezing, chronic disease, fatigue, and age, which are clinically recognized indicators of lung cancer risk. It is a vital preprocessing step in ML that involves selecting a subset of relevant features from a larger set of variables. It helps in enhancing model accuracy, reducing overfitting and lowering computational cost. This is especially crucial in lung cancer detection, where medical datasets often have high dimensionality. Selecting the most relevant features ensures that the model focuses only on the most informative traits.

The use of metaheuristic algorithms in this study is important because they provide an intelligent and automated way to select the most relevant clinical features from the dataset. Unlike traditional feature selection methods, metaheuristic approaches efficiently explore large search spaces and avoid local optima, which helps in identifying optimal feature subsets. This leads to improved classification accuracy, reduced overfitting, lower computational complexity, and enhanced model generalization. Therefore, integrating metaheuristic optimization strengthens the reliability and diagnostic performance of the proposed lung cancer classification pipeline.

Red Deer Optimization (RDO) is a nature-inspired metaheuristic algorithm mimicking the mating behavior and competition strategies of red deer. It categorizes the population into commanders, stags and hinds, simulating leadership and mating competition. It has key phases such as the Roaring Phase, in which male deer (solution candidates) try to increase fitness, the Fighting Phase, in which commanders compete with stags for dominance, and Harem Formation and Mating, in which best-fit males mate with females to generate offspring. Mathematically, it is defined as follows: let X_i_  ∈Rn be a feature subset in Roaring Exploration [44].

X_i_ = X_i_ +α·r_1_ − β·r_2_

where r_1,_ r_2_ ∈ [0, 1] and α, β are constant. In fighting selection, evaluate fitness f(x) and update based on best fights; the mating exploitation is offspring

C_ij_ = γ⋅x_i_ + (1 − γ)⋅x_j_

where γ ∈ [0, 1], x_i_ is a commander, and x_j_ a hind.

In Binary Grasshopper Optimization (BGO), the position update of each grasshopper is governed by the social interaction component, denoted as $S_{i}$ , which represents the cumulative effect of attraction and repulsion forces among individuals. The updated position of the i -th grasshopper is derived from this interaction term. The social interaction vector $S_{i}$ is defined as:

$$S_{i}=\sum_{j=1,j\neq i}^{N}s(d_{ij})\;{\hat{d}}_{ij}$$

where $d_{ij}$ represents the Euclidean distance between the i -th and j -th grasshoppers, and ${\hat{d}}_{ij}$ is the corresponding unit direction vector. The function $s(d_{ij})$ models the attraction–repulsion behavior and is defined as:

$$s(d)=fe^{-d/l}-e^{-d}$$

where f denotes the intensity of attraction, and l is the attractive length scale. This formulation ensures that nearby grasshoppers repel each other while distant ones attract, maintaining a balance between exploration and exploitation. The resulting continuous position values are then mapped to binary space using a sigmoid transfer function, enabling feature selection in the optimization process.

Gray Wolf Optimization (GWO) is a swarm intelligence algorithm inspired by the leadership hierarchy and hunting behavior of gray wolves. In lung cancer diagnosis, GWO is used to select optimal features from medical datasets (e.g., radiomics or gene expression data), which improves the performance of classifiers by reducing irrelevant or redundant features. In GWO, the population is divided into four types: alpha (*α*), beta (*β*), delta (*δ*) and omega (ω), with *α*, *β* and *δ* guiding the search. The position of a search agent (feature subset) is updated based on the top three wolves using

$$X_{\mathrm{new}}=\frac{X\alpha +X\beta +X\delta }{3}$$

X_new_ = Updated position of current search agent (solution).
*Xα* = Position of the alpha wolf (best solution found so far).
*Xβ* = Position of beta wolf (second-best solution).
*Xδ* = Position of delta wolf (third-best solution).

Division by 3 = takes the average of the three top wolves to update the current position.

Bee Colony Optimization (BCO) is a binary version of the Cuckoo Optimization Algorithm, inspired by the brood parasitism behavior of cuckoo birds. It is used for feature selection and classification tasks like lung cancer diagnosis, where each solution is a binary vector representing selected features. Mathematically it can be defined as

$$X_{i}\;t+1=\left\{\begin{array}{c} 1,\;\mathrm{if}\;p>r\;\mathrm{and}()\\ 0,\;otherwise \end{array}\right.$$

#### Model Evaluation Using Various Matrices

To assess the performance of applied machine learning models, several standard evaluation metrics were employed:

Accuracy: The ratio of correctly predicted samples to the total number of samples.

$$\mathrm{Accuracy}:\;\frac{TP+TN}{TP+TN+FP+FN}$$

Precision: The proportion of true positive predictions among all positive predictions. It measures how many of the predicted malignant cases are actually malignant.

$$\mathrm{Precision}:\;\frac{TP}{TP+FP}$$

Recall: The proportion of true positives correctly identified. It measures how effectively the model detects malignant cases.

$$\mathrm{Recall}:\;\frac{TP}{TP+FN}$$

F1-Score: The harmonic mean of precision and recall, providing a balance between the two.

$$F1\text{-}\mathrm{Score}:\;\frac{2\times (Precision\times Recall)}{Precision+Recall}$$

Weighted Average: Since the dataset may be imbalanced, the weighted average of precision, recall, and F1-score was also computed. It assigns weights proportional to class support number of instances per class, ensuring minority-class performance.

### 3.5. Computational Complexity Analysis

The computational complexity of the proposed framework depends on both the metaheuristic-based feature selection and the classifier training stages. For feature selection, the time complexity is approximately O (P × I × D × C), where P is population size, I is number of iterations, D is number of features, and C is classifier training cost. For classification models, Logistic Regression has complexity O(n·d) per iteration, and Random Forest and Gradient Boosting have complexity approximately O (T·n·d·log n), where n is number of samples, d number of features, and T number of trees. Given the relatively small dataset size (309 samples, 11 features), the overall computational cost remains moderate and suitable for clinical decision-support applications.

## 4. Results and Discussion

The experimental evaluation demonstrates that the proposed optimized pipeline achieved significant improvements in lung cancer classification. Among all the models, LR achieved the highest accuracy of 91.07%, followed by Gradient Boosting (87.5%) and Random Forest (87.5%), while SVC, KNN, and GNB reached 78.57%. These findings confirm that applying feature selection with metaheuristic optimization and hyperparameter tuning enhances predictive performance compared to baseline models. Performance was further analyzed using precision, recall, F1-score and weighted average, which demonstrated consistent results across classifiers. LR maintained balanced values across all metrics, reflecting both sensitivity (recall) and reliability (precision). Ensemble methods like RFC and GBC showed competitive accuracy but slightly lower generalization compared to LR. Simpler classifiers such as KNN and GNB produced modest results but remained computationally efficient. Logistic Regression achieved the highest accuracy (91.07%) and AUC (0.91), outperforming more complex ensemble models. This can be attributed to the relatively small dataset size and the structured, low-dimensional nature of the clinical features. Under such conditions, simpler linear models often generalize better and are less prone to overfitting than high-capacity ensemble classifiers.

**Figure 3:**
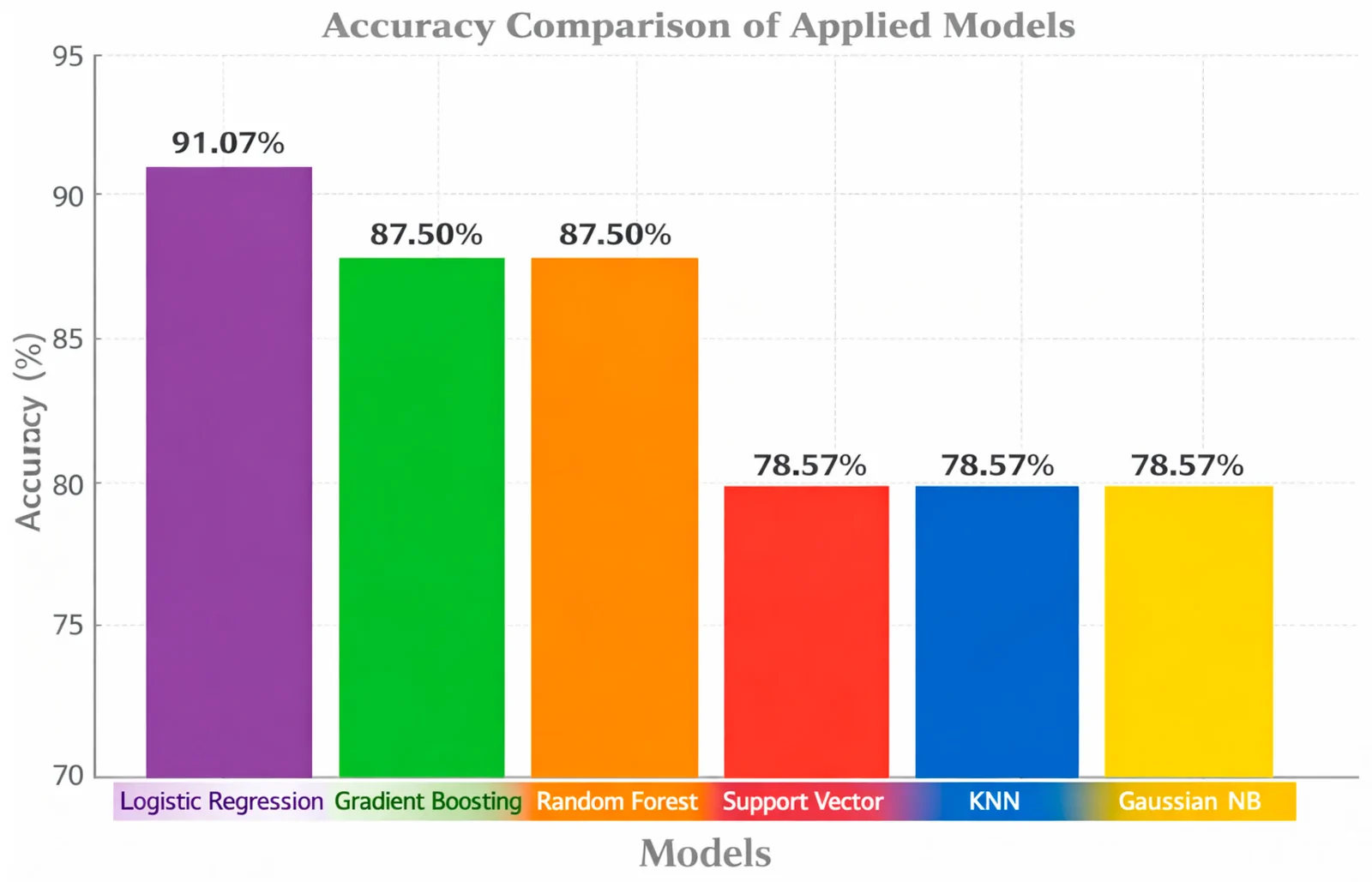
Comparison of applied models.

**Figure 4:**
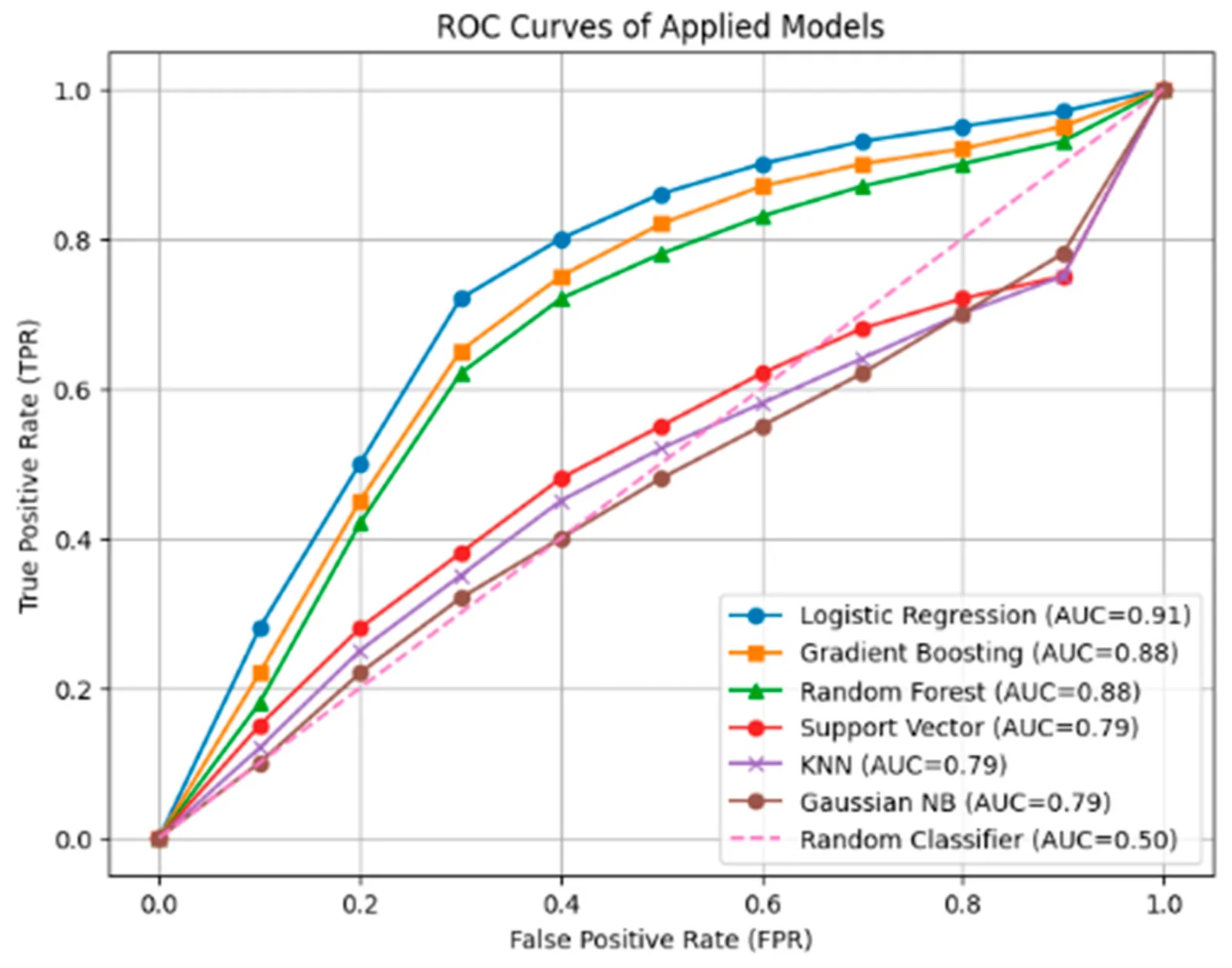
ROC curves of models.

Figure 3 illustrates the comparative accuracy of six machine learning classifiers applied to the lung cancer dataset. Logistic Regression (91.07%) achieved the highest accuracy, outperforming ensemble methods such as Gradient Boosting (87.5%) and Random Forest (87.5%). Simpler models like Support Vector Classifier (SVC), K-Nearest Neighbors (KNNs), and Gaussian Naïve Bayes (GNB) showed comparatively lower accuracies at 78.57% each. This result highlights that, while ensemble methods provide competitive performance, Logistic Regression offered the most reliable and interpretable outcome for this study. Figure 4 presents the Receiver Operating Characteristic (ROC) curves for the six applied machine learning classifiers. Logistic Regression achieved the highest AUC (0.91), followed closely by Gradient Boosting (0.88) and Random Forest (0.88), indicating strong discriminative power. In contrast, Support Vector Classifier (0.79), KNN (0.79), and Gaussian Naïve Bayes (0.79) produced lower AUC values, showing weaker performance in distinguishing between positive and negative cases. The diagonal dashed line represents a random classifier (AUC = 0.50), serving as a baseline. These results confirm that Logistic Regression consistently outperformed other models in terms of classification reliability. Although Gradient Boosting and Random Forest are capable of modeling non-linear relationships, their performance did not exceed Logistic Regression in this study. This suggests that the underlying feature outcome relationships in the dataset are largely linear and that additional model complexity does not necessarily translate into improved diagnostic performance when data size is limited.

To evaluate the effectiveness of the proposed optimized pipeline, its performance was compared with existing lung cancer prediction models reported in the literature. As summarized in Table 4, many previous approaches achieved accuracies ranging from approximately 68% to 89%, often relying on imaging data or single optimization strategies. In contrast, the proposed framework achieved an accuracy of 91.07% and an AUC of 0.91 using only routine clinical and lifestyle attributes, demonstrating competitive or superior performance with reduced model complexity and improved interpretability.

**Figure 5:**
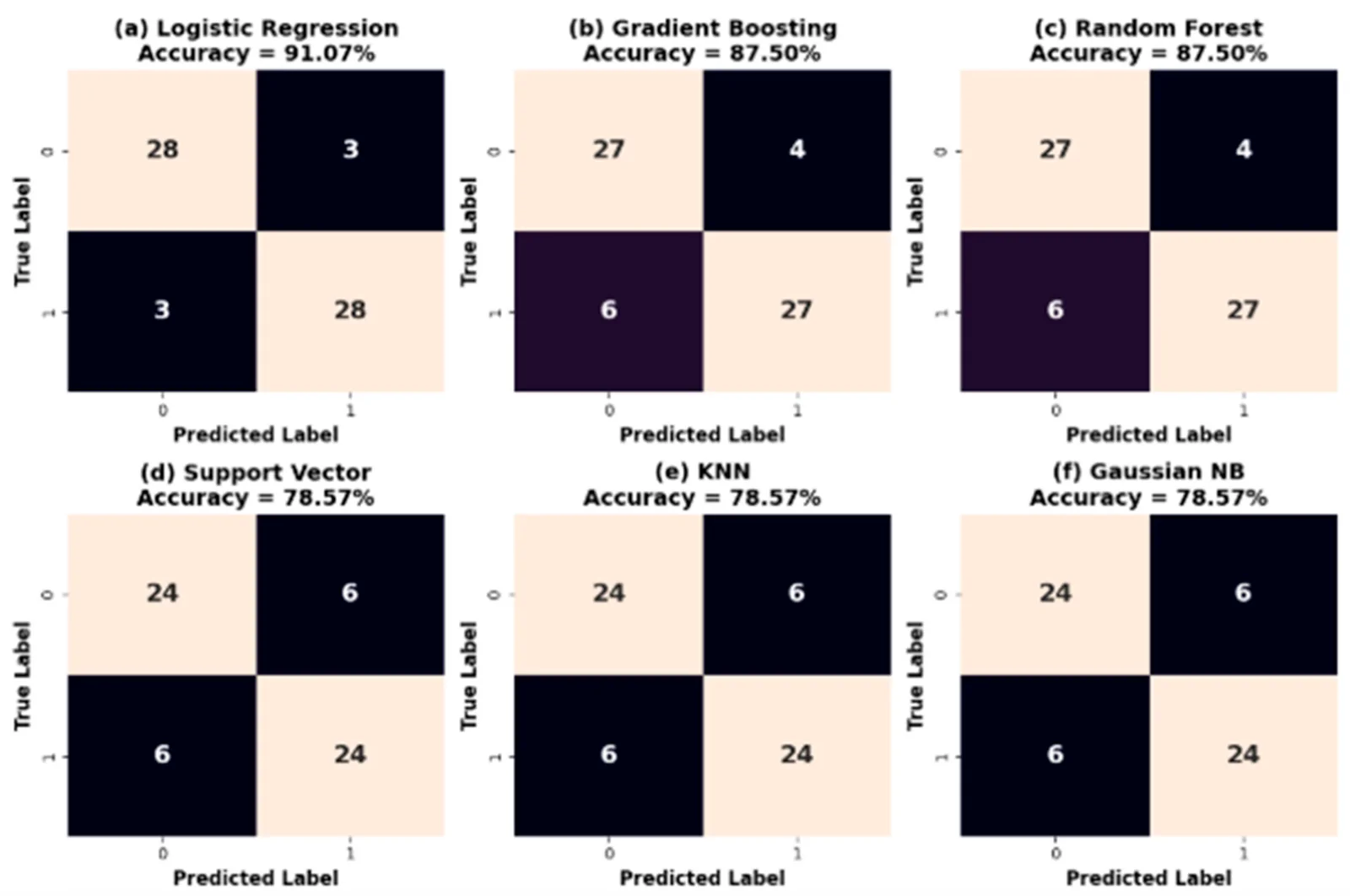
Confusion matrix.

In clinical decision-making, outcome misclassification has unequal consequences. False negative predictions are particularly critical in lung cancer diagnosis, as they may delay referral, imaging, and treatment, directly affecting patient prognosis. False positives, while less dangerous, may lead to unnecessary anxiety and additional diagnostic procedures. Therefore, reliance on accuracy alone may be misleading, especially in datasets with class imbalance. Class imbalance can bias model development by favoring the majority class during training, resulting in inflated accuracy despite poor sensitivity to malignant cases. To address this, performance evaluation in this study emphasizes recall, F1-score, ROC–AUC, and weighted averages, which better reflect minority-class performance. These metrics provide a more clinically relevant assessment and reduce the risk of deploying models that appear accurate but fail to reliably detect high-risk patients. Careful interpretation of these measures is essential to ensure that model outputs support safe and effective clinical decision-making. The Receiver Operating Characteristic (ROC) analysis provides insight into the discriminative capability of the applied classifiers across different decision thresholds. The Area Under the ROC Curve (AUC) quantifies how effectively a model distinguishes between malignant and benign lung cancer cases, independent of class distribution. An AUC value closer to 1.0 indicates strong classification performance, while a value of 0.5 represents random guessing. In this study, Logistic Regression achieved the highest AUC value of 0.91, demonstrating excellent discriminatory power and a high probability of correctly ranking malignant cases above benign ones. Gradient Boosting and Random Forest achieved AUC values of 0.88, indicating strong but slightly lower discrimination. In contrast, SVC, KNN, and Gaussian Naïve Bayes showed moderate AUC values of 0.79, reflecting comparatively weaker separation capability. From a clinical perspective, the high AUC of the Logistic Regression model suggests reliable performance for early lung cancer risk screening, where maintaining a balance between sensitivity and specificity is critical for reducing missed diagnoses while limiting false positives. Figure 5 presents the confusion matrices for all six applied classifiers, LR, GBC, RFC, SVC, KNN and GNB, showing the distribution of true positive, true negative, false positive and false negative predictions. Logistic Regression demonstrates the best balance, with the highest number of correctly classified cases, reflecting its superior accuracy (91.07%). Ensemble methods such as GBC and RFC also exhibit competitive classification performance, while SVC, KNN, and GNB display comparatively higher misclassification rates. These results confirm that LR is the most effective and reliable classifier for this dataset.

From a clinical perspective, the observed accuracy of 91.07% and AUC of 0.91 indicate strong discriminative ability, suggesting that the model can reliably distinguish high-risk from low-risk individuals. In routine practice, such performance may increase clinician confidence during early screening, support timely referral for confirmatory imaging in high-risk patients, and reduce unnecessary investigations among low-risk cases, thereby improving early detection efficiency. From a clinical perspective, false negatives represent the most critical error, as missed malignant cases may delay diagnosis and treatment. The confusion matrix analysis shows that Logistic Regression produces the lowest false negative rate among the evaluated models, which is consistent with its higher recall and AUC values. This indicates a stronger ability to correctly identify malignant cases, reducing the risk of missed diagnoses. Ensemble models exhibited slightly higher false negative counts, suggesting a trade-off between overall accuracy and sensitivity. Emphasizing recall and false negative minimization is therefore essential when considering deployment in early lung cancer risk screening. Despite promising results, performance estimates are derived from a single dataset without external validation. Therefore, the observed improvements should be interpreted as exploratory rather than definitive evidence of clinical superiority. Although the proposed optimized machine learning pipeline demonstrates encouraging performance, the achieved results are not yet optimal. This limitation is primarily attributed to the relatively small dataset size and the absence of external validation. Consequently, the findings should be interpreted with caution and are best regarded as exploratory or a proof-of-concept rather than definitive evidence of clinical applicability. Further improvements are expected with larger, multi-center datasets and additional validation strategies.

The results demonstrate that the proposed optimized machine learning pipeline can effectively distinguish between malignant and benign lung cancer cases using routine clinical and lifestyle data. Logistic Regression achieved the highest accuracy (91.07%) and AUC (0.91), indicating strong discriminative capability and stable generalization. This behavior is consistent with the dataset characteristics, including limited sample size and predominantly binary clinical features, where simpler linear models are less prone to overfitting than high-capacity ensemble methods. From a clinical safety perspective, outcome misclassification carries unequal consequences. False negative predictions are particularly critical in lung cancer diagnosis, as missed malignant cases may delay referral, imaging, and treatment, adversely affecting patient outcomes. The confusion matrix analysis shows that Logistic Regression produced fewer false negatives compared to other classifiers, aligning with its higher recall and supporting its suitability for early risk screening where sensitivity is prioritized.

Class imbalance further influences both model development and evaluation. In such settings, accuracy alone may be misleading, as models may favor the majority class while underperforming on malignant cases. To address this bias, recall, F1-score, weighted metrics, and ROC–AUC were emphasized to ensure clinically meaningful evaluation. These measures provide a more reliable basis for clinical decision-making by reducing the risk of overlooking high-risk patients.

Despite the encouraging results, several limitations should be acknowledged. The study is based on a single dataset with a relatively small sample size and lacks external validation, which may limit generalizability. Additionally, performance gains from metaheuristic feature selection should be interpreted as exploratory rather than statistically definitive. Future work should include validation on larger multi-center datasets, the integration of explainable AI techniques, and prospective clinical evaluation to further assess safety and applicability.

## 5. Conclusions and Future Work

This study proposed an optimized machine learning pipeline for lung cancer classification by integrating feature selection through metaheuristic algorithms and hyperparameter tuning. Among the applied models, Logistic Regression (LR) demonstrated the highest accuracy (91.07%), followed by Gradient Boosting (GB) and Random Forest (87.5%), while Support Vector Classification (SVC), K-Nearest Neighbors (KNN), and Gaussian Naïve Bayes (GNB) achieved moderate performance (78.57%). The findings indicate that the joint application of advanced feature selection techniques and systematic hyperparameter optimization substantially enhances model generalization capability, predictive accuracy, and diagnostic reliability in clinical decision-support systems. Despite the promising performance of the proposed pipeline, the findings should be interpreted with caution. The study is based on a relatively limited dataset and lacks external validation on independent clinical cohorts. Therefore, the results are best regarded as exploratory or a proof-of-concept rather than definitive evidence of clinical utility. Further validation on larger, multi-center datasets is required before clinical deployment.

For future work, the proposed framework can be extended by integrating advanced deep learning architectures, such as CNNs and RNNs, to jointly analyze medical imaging data and structured clinical attributes within a multimodal learning paradigm. Incorporating Explainable AI (XAI) techniques will further enhance model transparency and interpretability, enabling clinicians to better understand and validate decision outcomes. Additionally, expanding the dataset to include more diverse clinical records and heterogeneous multimodal sources will improve generalization capability and robustness, facilitating reliable real-world clinical deployment.

## Figures and Tables

**Table 1:** Shows the comparison.

Research Work	Dataset	Method Used	Results
[36]	Custom dataset with clinical risk factors	Bagging, Randomized Node Optimization	RF achieved 68% accuracy
[37]	SEER database	AdaBoost, Bagging, Ensemble methods	Accuracy ranged from 75 to 85%
[38]	Imbalanced clinical dataset	RF with Bagging Ensemble	Reported 84.3% accuracy
[39]	Mixed-quality clinical samples	Random Forest + Self-paced Learning Bootstrap	Accuracy approx. 86.1%
[40]	Clinical dataset (used for cervical/lung)	Voting Ensemble (RF + MLP), PCA, Select Best, XGB	Best model achieved 79% accuracy
[2]	CRCB—West China Hospital	Optimized XGB (EXSA), Cox, RSF	Best result 87.2% accuracy
[41]	Public lung cancer dataset	XGB, LGBM, Bagging, AdaBoost, etc.	Most < 89%; XGB reached 94.42%, others lower
Proposed Model (This Study)	Kaggle + Lanzhou University + Clinical Data	LR, SVC, GBC, RFC, GNB, KNC	LR and GNB achieved highest accuracy of 91.07%.

**Table 2:** Dataset attributes for lung cancer classification.

Attribute	Description	Values
Gender	Biological sex of the patient	0 = Female, 1 = Male
Age	Age of the patient in years	Numeric (e.g., 20–80)
Smoking	History and intensity of tobacco use	0 = No, 1 = Yes
Yellow Fingers	Presence of nicotine stains on fingers	0 = No, 1 = Yes
Anxiety	Reported stress or anxiety levels	0 = No, 1 = Yes
Peer Pressure	Social influence towards smoking or drinking	0 = No, 1 = Yes
Chronic Disease	Presence of long-term diseases (e.g., COPD, asthma)	0 = No, 1 = Yes
Fatigue	Persistent tiredness or weakness	0 = No, 1 = Yes
Allergy	History of allergic reactions	0 = No, 1 = Yes
Wheezing	Abnormal breathing sounds indicating lung dysfunction	0 = No, 1 = Yes
Alcohol Consuming	Frequency of alcohol intake	0 = No, 1 = Yes
Class (Target)	Lung cancer diagnosis	0 = Benign, 1 = Malignant

**Table 3:** Selected features and clinical relevance.

Feature	Clinical Significance
Smoking	Major risk factor
Wheezing	Indicates lung dysfunction
Chronic Disease	Associated with respiratory vulnerability
Fatigue	Common symptom in malignancy
Age	Higher risk in older adults

**Table 4:** Comparison of the proposed model with existing lung cancer prediction approaches.

Ref.	Dataset Type	Method	Accuracy/AUC
[45]	Clinical	RF + Bagging	68%
[46]	SEER	Ensemble Models	75–85%
[47]	Public Dataset	XGBoost	94.42%
Proposed Model	Clinical and Lifestyle	Optimized ML Pipeline	91.07%/0.91

## Data Availability

The data supporting the findings of this study are publicly available. This dataset contains clinical information related to patients with symptoms or diagnosis of lung cancer. It is intended for research and machine learning applications focused on early detection and prediction of lung cancer all of which are available on Kaggle (https://www.kaggle.com/datasets/uciml/breast-cancer-wisconsin-data, accessed on 26 August 2025).
